# Versorgung und Versorgungskonzepte in der Kinder- und Jugendpsychiatrie

**DOI:** 10.1007/s40211-022-00435-y

**Published:** 2022-10-26

**Authors:** Leonhard Thun-Hohenstein

**Affiliations:** Paracelsus Medizinischen Privatuniversität, Salzburg, Österreich

**Keywords:** Versorgung, Kinder- und Jugendpsychiatrie, Versorgungskonzepte, Versorgungsplanung, CAP-Care, Child, And adolescent psychiatry (CAP), Care concepts, Care planning

## Abstract

**Hintergrund:**

Die Situation der Kinder- und Jugendpsychiatrischen Versorgung hat sich seit 2007, seit der Schaffung des Sonderfaches KJP+P, sukzessive verbessert. Die Arbeitsgruppe Versorgung der ÖGKJP gibt in diesem Heft einen Überblick über die KJP-Versorgungssituation in Österreich. In dieser Einführung geht es um die Darstellung der rechtlichen Vorgaben und deren Umsetzung. Es bestehen nach wie vor massive Defizite der KJP-Versorgung.

Im Österreichischen Strukturplan Gesundheit (ÖSG) ist das momentan gültige Versorgungskonzept festgeschrieben und umfasst die ambulante, teilstationäre und stationäre Versorgung. Für die ambulante und stationäre Versorgung existieren Messzahlen, für die teilstationäre Versorgung nicht.

Das Fachgebiet KJP hat verschiedene Besonderheiten: die Multimodalität, die Multiprofessionalität, die Altersgruppen der Betroffenen, pro Alter unterschiedliche und unterschiedlich ausgeprägte Erkrankungen, die Beschränkung auf eine bestimmte Altersgruppe (< 19 Jahre) und – das verbindet es mit dem Fach Psychiatrie – eine hohe Prävalenz der Erkrankungen sowie die Integration der Psychotherapeutischen Medizin in den Facharzt.

**Schlussfolgerungen:**

Die aus diesen Besonderheiten sich ergebenden Konsequenzen – altersdifferenzierte Angebote, fachübergreifende Kooperation etc. – haben bisher keinen Eingang in die strukturellen und finanziellen Berechnungen gefunden. Anhand der den Begriff Versorgung beschreibenden Begriffe: „care“, „provision“ und „supply“ wird ein Modell vorgestellt, das bei der Planung von Gesundheitsmaßnahmen in diesem Bereich als Denkansatz herangezogen werden könnte. Da psychisch kranke Kinder und Jugendliche sich in allen Teilen der Gesellschaft finden, wird die Einrichtung einer zentral verantwortlichen und ressortübergreifenden Stelle für Mental Health gefordert.

## Abstract (en)

**Background:**

The situation of child and adolescent psychiatric care has gradually improved since 2007, since the creation of the special medical specialty CAP+P. The workinggroup “Versorgung” of the OGKJP provides an overview of the CAP-Services in Austria. There are still massive deficits in supply. This introduction deals with the presentation of the legal requirements and their implementation.

The currently valid care concept is laid down in the Austrian Structure Plan Health (ÖSG) and includes outpatient, day-care and inpatient care. Regulatory structures exist for outpatient and inpatient care, but not for dayclinic care.

The field of CAP has various special features: the multimodality, the multiprofessionalism, the age groups of those affected, different and differently presented diseases per age, the restriction to a certain age group (< 19 years) and—this connects it with adult psychiatry—a high prevalence of the diseases as well as the integration of psychotherapeutic medicine into the specialization. The consequences resulting from these special features have not yet found their way into the structural and financial calculations.

**Conclusions:**

Based on the terms describing the term care: “care”, “provision” and “supply”, a model is presented that could be used as a way of thinking when planning health measures in this area. Since mentally ill children and adolescents can be found in all parts of society, the establishment of a centrally responsible and interdepartmental body for mental health is required.

## Einleitung

Das Sonderfach Kinder und Jugendpsychiatrie (KJP) besteht nun bereits seit 2007 und wurde seitens der Bundesregierung 2015 [1] neugeregelt und in der Ärzteausbildungsordnung als Fachärzt*in für Kinder- und Jugendpsychiatrie, Psychosomatik und psychotherapeutische Medizin definitorisch festgehalten. Eine detaillierte Beschreibung der Entwicklung der KJP in Österreich findet sich im Sonderheft „10 Jahre Fach Kinder- und Jugendpsychiatrie“ [2].

Obwohl bereits seit 1997 [2] als Kinder- + Jugendneuropsychiatrie in die Leistungsorientierte Krankenhausfinanzierung (LKF) aufgenommen, zog erst die Schaffung des eigenen Sonderfaches 2007 einen eigenen für dieses Fach geltenden, allgemeinen Versorgungsauftrag im Österreichischen Strukturplan Gesundheit (ÖSG) nach sich. Der ÖSG gilt als „*das zentrale Planungsinstrument auf Bundesebene für die integrative Versorgungsplanung in Österreich und“ *ist* „seit 2013 integraler Bestandteil der Zielsteuerung-Gesundheit. Er enthält als Rahmenplan verbindliche Vorgaben für die Planung bestimmter Bereiche des Gesundheitsversorgungsystems sowie Kriterien für die Gewährleistung der bundesweit einheitlichen Versorgungsqualität. Mit dem ÖSG wird sichergestellt, dass Gesundheitsversorgung in Österreich ausgewogen verteilt und gut erreichbar ist und in vergleichbarer Qualität auf hohem Niveau angeboten wird“* [3]. Der ÖSG wird von der Bundeszielsteuerungskommission herausgegeben.

Der ÖSG ist also das Instrument der Versorgungsplanung für das österreichische Gesundheitssystem, also auch für das Sonderfach KJP und „*durch die Vereinbarung österreichweiter Versorgungsstandards sollen die in einzelnen Versorgungsbereichen bestehende Über‑, Unter- oder Fehlversorgung der Bevölkerung hintangehalten und eine entsprechende Qualität der Versorgung sichergestellt werden“* [4]. In diesem Sonderheft werden die Vorgaben des ÖSG in verschiedenen Bereichen der Kinder- und Jugendpsychiatrie als Grundlage der Diskussion herangezogen.

Versorgungsforschung ist ein wichtiger Teil der Gesundheitssystemforschung und beschäftigt sich in erster Linie mit der Organisation, der Steuerung und Finanzierung des Kranken- und Gesundheitswesens. Versorgung wird in diesem Zusammenhang als die Bedienung relevanter Bedürfnisse oder Defizite von Lebewesen bezeichnet, die Bedienung mit medizinischen Leistungen als Gesundheitsversorgung. Ganz zentral wird bei Erbringung von Gesundheitsleistungen der Ort der Versorgung bzw. der Erbringung der Leistung berücksichtigt. Das Österreichische Bundesinstitut für Gesundheitswesen hat 2004 [5] und die Österreichische Gesellschaft für Kinder- und Jugendpsychiatrie 2006 [6] haben im Rahmen der Vorbereitung des neuen Sonderfaches die Gesundheitsversorgung durch die Fachärzt*innen für KJPP beschrieben und insbesondere die verschiedenen Versorgungsebenen (siehe Tab. 1) definiert.

**Table 1:** 

Versorgungsniveau	Einrichtungen	Finanzierung
Stationär	Abteilungen für KJP	LKF
Konsiliar- und Liäsondienst	Abteilungen für KJP;	LKF
	Psychosomatische Einrichtungen	LKF
	Niedergelassene FÄ	Verschieden
Teilstationär	Abteilungen für KJP	LKF
	Extramurale Tageskliniken	Verschieden
Ambulant	Abteilungen für KJP	LKF
	Ambulatorien	Verschieden
Mobile Einheiten	–	–
Dezentrale gemeindenahe Netzwerke	–	–
Niedergelassener Bereich	Facharztpraxen	Krankenkassen

**Figure 1:**
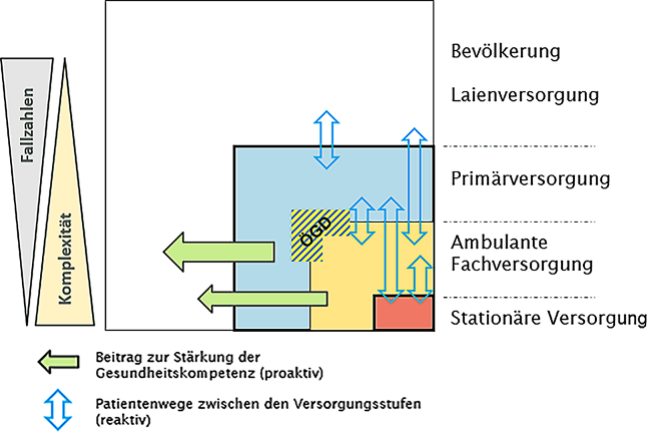

Zuletzt wurden diese Arbeiten durch Fliedl et al. [7] aktualisiert. In Abb. 1 ist das vom ÖSG definierte Versorgungsmodell dargestellt.

Der ÖSG 2021 [8] definiert die Anforderungen an eine qualitätvolle Versorgungsplanung wie folgt: es geht um die „*Sicherstellung des offenen Patientenzugangs zum evidenzgesicherten ****medizinischen Fortschritt****. Die Sicherstellung von ****fachlicher Expertise**** in den Behandlungsteams (ÄrztInnen und Angehörige pflegerischer und therapeutischer Gesundheitsberufe) durch Qualifikation (Aus‑, Fort- und Weiterbildung) und Routine“ *weiters um die „*Einhaltung der im ÖSG enthaltenen ****Qualitätskriterien**** und Einhaltung ****ökonomischer Grundprinzipien**** im Hinblick auf ausreichende Leistungsmengen, Fixkostendegression und Nutzungsgrad von eingesetzten Ressourcen und Kapazitäten ohne Qualitätseinbußen“ *(Hervorhebungen lt. Originaltext)*.*

Die Fragen, die sich in diesem Zusammenhang stellen, sind: wer definiert in unserem Fach was „medizinischer Fortschritt“ ist und welche Qualitätskriterien werden zur Beurteilung und Planung herangezogen und wie wirken sich ökonomische Grundprinzipien auf diese Planungen aus? Weiters wie fließen diese Bewertungen in die Versorgungsaufträge?

**Figure 2:**
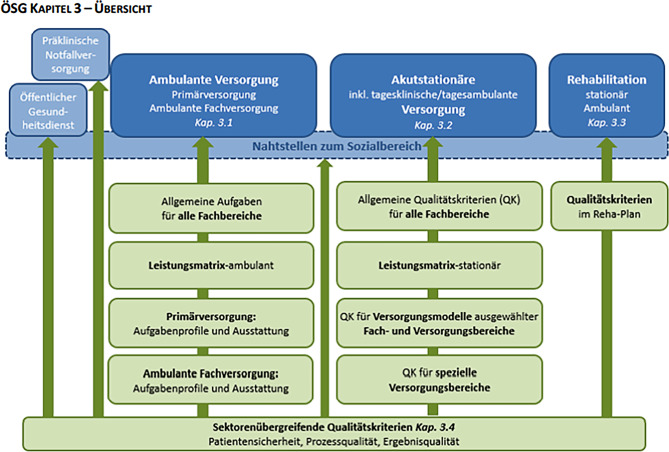

Wie sieht nun die – zumindest theoretische Umsetzung der Versorgung (Abb. 2) für unser Fach aus?

Der ÖGD (Öffentlicher Gesundheitsdienst) ist in unserem Fachbereich im Wesentlichen für die Begutachtung nach dem Unterbringungsgesetz im ambulanten Bereich zuständig, dies in enger Kooperation mit Rettung und Polizei (Präklinische Notfallversorgung). Die ambulante Versorgung umfasst nach dem ÖSG die Bereiche Primärversorgung, in dem bisher die Beteiligung der Kinder- und Jugendpsychiatrie seitens der Gesundheitsplanung nicht vorgesehen ist, obwohl es dafür gute Sach- und Fachbezogene Argumente gibt [9]. In die sogenannte Ambulante Fachversorgung fallen Einzel- und Gruppenpraxen sowie die selbstständigen Ambulatorien, Miniambulatorien (siehe auch Stellungnahme des ÖGKJP-Vorstandes [10]) und die, an klinische Abteilungen angeschlossenen Fachambulanzen. Laut ÖSG sollen neben den Allgemeinen Aufgaben für alle Fachbereiche eine auf das Fach hin abgestimmte Leistungsmatrix, Aufgabenprofile und Ausstattungskriterien definiert werden. Diese Definition ist bisher nicht erfolgt und wird vor allem aufgrund des Föderalismus in den einzelnen Bundesländern höchst unterschiedlich wahrgenommen. Der ÖSG hat auch Richtwerte zur ambulanten Versorgung angeführt, es sollten demnach 0,6–1,2 Ärztlich Ambulante KJP-Versorgungseinheiten (ÄAVE; entspricht ärztlichen Vollzeitäquivalenten [11]) /100.000 EW die Bevölkerung versorgen.

Die nächste Ebene der Versorgung sind Tagesklinische Einrichtungen, die im ÖSG 2017 noch zu den stationären Einrichtungen gezählt werden, nach LKF aber bereits seit einigen Jahren als ambulante Einrichtungen geführt werden.

Im stationären Bereich wird unterschieden je nach Status (Standard, Schwerpunkt, Zentralkrankenanstalt) der Krankenanstalt, an der die jeweilige Abteilung für KJP angesiedelt ist. Insgesamt existieren in Österreich zurzeit 13 Abteilungen für KJP, davon sind mit Stand 30.4. insgesamt fünf Abteilungen für KJP an Universitätskliniken integriert und somit automatisch Teil einer Zentralkrankenanstalt. In der Klassifikation der Krankenanstalten in Österreich [12] werden psychiatrische Abteilungen oder Kinder- und Jugendabteilungen als „Spezialversorgung“ gesehen und sind somit per se Schwerpunktkrankenanstalten. Referenzzentren (RFZ) oder hochspezifische Spezialzentren im engeren Sinne (lt.ÖSG) gibt es in Österreich im Fach KJP (noch) nicht. Vorgaben bezüglich des Umfangs der Versorgung sind definiert (z. B.: Bettenmessziffer BMZ zwischen 0,6–1,1 Betten auf 100.000 EW oder auch bestimmte Personalstrukturkriterien). Auch für die stationären Einrichtungen gibt es keine definierte Kooperationsformen – zum Beispiel ist der gesamte KJP-Konsiliar- und Liäsondienst (v. a. in die Pädiatrie, Psychiatrie und Kinder- und Jugendhilfe) nicht definiert, einige Zentralspitäler schaffen die Möglichkeiten von sogenannten Interdisziplinären Schwerpunkten, die aber aufgrund der fehlenden Abbildung in ÖSG und LKF nicht leicht umzusetzen sind.

In den letzten Jahren gab es einige Aktivitäten seitens der Politik (Kinder- und Jugendgesundheitsstrategie, Nationaler Aktionsplan etc.), die sich aber mit der Erstellung von Berichten und Feststellen der Defizits begnügt haben. Die wesentlichste Veränderung war die Erweiterung der Mangelfachregelung auf einen neuen Ausbildungsschlüssel von 1 Facharzt:in auf 2 Ausbildungsärzt:innen.

Das ist die politische, gesetzgeberische und auch finanzpolitische Sicht auf die KJP-Versorgung, doch könnte und sollte Versorgung auch von Seiten der Inanspruchnahme-Population aus betrachtet werden. Zumeist wird dabei von epidemiologischen Daten ausgegangen bzw. auch von einzelnen Erkrankungen und ihrer Versorgungsnotwendigkeit. Epidemiologische Daten aus Österreich (MHAT-Studie [13]) weisen auf eine hohe Punktprävalenz psychischer Auffälligkeiten (23,9 %) hin, die durch die SARS-Cov-2-Pandemie noch deutlich angehoben wurde (40 % Steigerung der Inanspruchnahme [14], Zunahme psychopathologischer Auffälligkeiten Depression, Angst, Essstörungssymptomatik [15]). Die Inanspruchnahme war allerdings schon vor der Pandemie nicht ausreichend, lediglich 48 % gaben an sich Hilfe geholt zu haben, weitere 25 % wünschen sich Hilfe [13]. Eine Folge dieser mangelnden Inanspruchnahme ist das Ausweichen auf nicht-medizinische Angebote, das in erster Linie von wohlhabenderen Familien und von Familien mit Kindern, die an externalisierenden Störungen leiden, wahrgenommen wird. Versorgungsmängel führen somit zu zusätzlicher Benachteiligung ärmerer Betroffener [16].

Kinder- und Jugendlichen sind in allen Sektoren der Gesellschaft zu finden: Gesundheit, Schule, Soziales, Justiz, Wirtschaft und Familie. Es ist daher in all diesen Bereichen auf die Situation der psychisch wie körperlich kranken Kinder und Jugendlichen Rücksicht zu nehmen und planerisch wie präventiv tätig zu werden. In allen genannten Bereichen ist dies im Moment nur ansatzweise vorhanden und eine Kooperation dieser Bereiche miteinander ist kaum zu finden. Aus diesem Grunde ist, um dieser Materie auch nur annähernd gerecht zu werden, die Forderung nach einem eigenen Mental Health Ministerium dringend zu unterstützen, wie dies in Australien, Kanada, Großbritannien und Irland bereits umgesetzt ist.

Weitere Aspekte der Versorgung sind krankheitsspezifische Aspekte. Welche Erkrankung muss von wem, auf welcher Ebene der Versorgung, wie versorgt werden? Beispiel Anorexia nervosa: in der S3-Leitlinie der AWMF [17] ist festgehalten, dass als Mindeststandard zu gelten habe: Versorgung durch eine/n Kinder- und Jugendpsychiater:in gemeinsam mit Diätolog:in und Psychotherapeut:in, alle müssen Knowhow in der Behandlung von Anorexia haben. Diese Kriterien erfüllen in Österreich nur wenige der ambulanten Einrichtungen, im stationären Bereich ist die Situation noch dünner. Und dennoch wurde die medizinische Versorgung von PatientInnen mit Anorexie seitens des Gesundheitsministeriums in einer Beantwortung einer parlamentarischen Anfrage als ausgezeichnet beschrieben [18]. Diese unterschiedliche Wahrnehmung seitens der offiziell Verantwortlichen und der Betroffenen zieht sich eigentlich durch die gesamte Materie der Kinder- und Jugendpsychiatrie, Bespiele gäbe es genug: um nur einige zu nennen: Autismus-Versorgung, die Versorgung von straffällig gewordenen Jugendlichen, die Transition, minderjährige Flüchtlinge, Mobbing etc.

Neben der Krankheitsspezifischen Versorgung könnte man auch eine Altersspezifische Versorgung überlegen, da die psychischen Erkrankungen sich je nach Alter unterschiedlich manifestieren, zu unterschiedlichen Altern auftreten und entsprechend unterschiedliche Herangehensweisen in Diagnostik und Therapie benötigen. KJP-Versorgung sollte daher dort sein, wo die Kinder und Jugendlichen sind, an Kindergärten und Schulen, an allen Krankenanstalten, die Kinder und Jugendliche betreuen, in den Primärversorgungszentren und nahe bei den Kinder- und Jugendärztinnen – liegt doch das Risiko einer psychiatrischen Störung bei körperlich erkrankten oder behinderten Kindern und Jugendlichen nochmals einiges höher als bei den gesunden Kindern und Jugendlichen [19, 20]. Schlussendlich sollten die Fachärzt:innen für KJP in allen Einrichtungen, die Kinder und Jugendliche betreuen (Kinder- und Jugendhilfe, AMS, Justiz etc.) in enger Kooperation mit den Trägerorganisationen arbeiten können. Diese Versorgungsstrukturen müssen sich neben dem Alter an der Entwicklung orientieren und entsprechend personell und fachlich adäquat aufgestellt sein.

**Figure 3:**
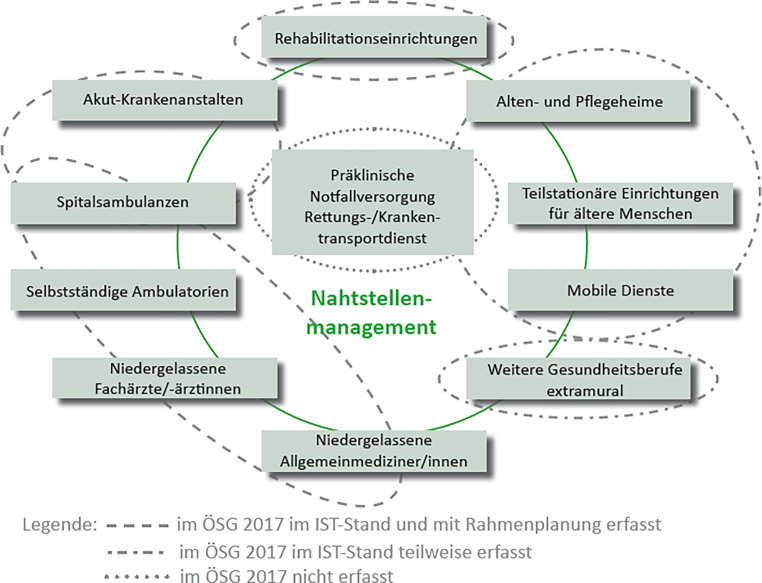

Kinder- und Jugendpsychiatrische Versorgung weist aber noch weitere Besonderheiten auf. Es ist neben der Psychiatrie das einzige Fach, das einen impliziten, nämlich in der Facharztdefinition integrierten, psychotherapeutischen Versorgungsauftrag beinhaltet, dem natürlich in Ausbildung, Lehre aber auch im Rahmen der Formulierung der Versorgungsaufträge, der Bereitstellung von Ressourcen und Finanzierung strukturell Rechnung getragen werden muss. Dies ist bisher nur ansatzweise erfolgt. Das Fach KJP ist DAS Fach, in dem Interdisziplinarität, Multimodalität und Kooperation zentral im Selbstverständnis des Faches enthalten sind [6]. Diese Themen werden im stationären Rahmen am ehesten umgesetzt, für den niedergelassenen Arzt ist es bisher schier unmöglich (wenngleich viele dies trotzdem umsetzen) dies in seine Tätigkeit zu integrieren, solange Kooperation, Multimodalität und -professionalität in den Planungs- und Finanzierungsrichtlinien der ÖGK und derer der Kooperationspartner (z. B. Österreichischen Ärztekammer) nicht vorkommen. Es gibt zwar Grafiken im ÖSG, die diese Kooperation strukturell darstellen (siehe Abb. 3), doch in den Finanzierungsmodellen scheinen die dazu nötigen Leistungen nicht als eigene Position auf.

Die Alterseinschränkung der KJP auf unter 19-Jährige ist aus KJP-Sicht fachlich nicht zu begründen, sondern stellt eine reine Machtpolitische Maßnahme dar, wäre sie nämlich inhaltlich begründet, nämlich mit der ausschließlichen Qualifizierung für Kinder und Jugendliche, dann dürfte – außer den Pädiater:innen und Allgemeinärzt:innen eigentlich kein anderes Fach Kinder- und Jugendliche behandeln. Dass dem nicht so ist, ist evident. Aus der wissenschaftlich-fachlichen Perspektive ist festzuhalten, dass die Entwicklung des Menschen nicht mit dem Erreichen des 19. Geburtstags abgeschlossen ist oder das Erwachsen-Sein genau dann beginnt. Mittlerweile ist es klar, dass für die Menschen zwischen 16 und 25 ähnliche Entwicklungsbedingungen gelten, ähnliche Krankheitsbilder sich zeigen und diese Menschen in der Regel sich noch in einem Entwicklungsmoratorium („Emerging Adulthood“) befinden, das sowohl Fachwissen aus dem KJP-Bereich und dem Erwachsenenbereich benötigt, die Strukturen der Versorgung aber eher an den Bedürfnissen eines jüngeren Klientels sich orientieren müssen. Der Gesetzgeber hat sich diese Themas bereits angenommen, in den Ausbildungsrichtlinien beider Fächer (KJP, Psychiatrie) ist die „Transitionspsychiatrie“ als Ausbildungsinhalt respektive eigenes Modul vorgesehen. Allerdings wird diesen Bedürfnissen der Patient*innen und der Ausbildung der Ärzt*innen auf der strukturellen Ebene nicht ausreichend Rechnung getragen. Neben dieser strukturellen Verbesserung sollte eine Ausweitung des Faches Kinder-Jugendpsychiatrie bis zum Alter von 24 oder 25 Jahren angedacht werden, damit diese jungen Menschen auch in das Knowhow von KJP-Betreuung kommen können.

Eine weitere Besonderheit ist die, im Vergleich mit allen übrigen medizinischen Fächern deutlich längere Verweildauer bei Spitalsbehandlungen, die der Tatsache geschuldet ist, dass psychische Erkrankungen ausreichend Zeit und entsprechende Rahmenbedingungen benötigen, um sich zu verändern. Damit steigt die Bedeutung der Qualität von architektonischen und Sicherheits-bezogenen Gegebenheiten, es verändert das Tagesangebot, die nötige Tagesstruktur, es hebt die Bedeutung der Gruppe der Mit-Patientinnen und deren Auswahl. Ebenfalls ist klar, dass derartig lange Aufenthalte nur unter bestimmten, der Behandlung zugrunde liegenden Konzepten erfolgen können. Kinder- und Jugendpsychiatrische stationäre Behandlung unter den genannten guten Bedingungen ist äußerst erfolgreich in der Reduktion der Aufnahmsymptomatik (Symptomreduktion) und Stabilisierung der Patientinnen auch in der nachstationären Zeit und es können ebenfalls signifikante Verbesserungen der Lebensqualität erzielt werden [21].

Sollte die Versorgungsplanung der Kinder- und Jugendpsychiatrie in den nächsten Jahren auf neue Beine gestellt werden, könnte man sich an einer anderen Begrifflichkeit des Versorgungsbegriffs orientieren: übersetzt man Versorgung ins Englische, wird man mit drei Begriffen konfrontiert: *Care, Supply, Provision*. Unter *care* würde Pflege, Sorgfalt, Betreuung verstanden werden können und sich damit im Rahmen der Versorgungsdiskussion mit folgende Fragen verbinden: was bekommt der versorgte Mensch tatsächlich, örtlich, zeitlich (Dauer, Frequenz), personell (welche Berufsgruppen?), qualitativ (ambulant vor stationär; berufliche Qualitätsstandards), supportiv (finanziell, sozial, Behandlung, tragendes Netzwerk etc.) und auf welcher Ebene der Versorgung? Sind die Angebote auf das Wohlbefinden der Kinder und Jugendlichen und ihrer Angehörigen abgestellt (z. B. Orientierung an Menschenrechten und Kinderschutzkriterien)? Wie ist die Qualitätssicherung auf dieser Ebene, Erfolgsrate, Patient:innenzufriedenheit, Mitarbeiter:innen-zufriedenheit?

Der zweite Begriff ist *provision*: diesen Begriff könnte man mit den Begriffen Bereitstellung, Vorkehrung, Beschaffung, Bestimmung verstehen und ist mit folgenden Fragen verbunden: wer ist für welche Zur-Verfügung-Stellung verantwortlich und zuständig (Stichwort z. B. One-Stop-Shop; Behandlungstechniken etc.) und was wird wie auf welcher Versorgungseben angeboten?

Der dritte Begriff in diesem Zusammenhang ist *supply*, für diesen Begriff gibt es ebenfalls mehrere Interpretationen oder Bedeutungen: Lieferung, Angebot, Zufuhr oder Zuführung. Die Lieferung und Bereitstellung der Leistung (wann, wo und wie) wiederum in Abhängigkeit vom Alter des Patient*in und vom Versorgungsniveau und dem Ort der Leistung. In Tab. 2 ist ein Beispiel für diese Herangehensweise anhand von Leitlinien und dem „Care,Provision,Supply“-Modell zur Versorgung von Menschen mit Essstörungen [17, 22] angeführt.

**Table 2:** 

	CARE	PROVISION	SUPPLY
Niedergelass. FA f. KJP	Erst-Gespräch/Früherkennung – Eventuell Screening FB (SCOFF)	Facharztpraxis Kassenvertrag (inkl Kooperation)	Wie viele KJP-FÄ/Region/EW?
		Evidenzbasiertes Knowhow für Essstörungsbehandlung in der Praxis	Zeit
	Diagnosestellung – Symptomspezifisch (z. B. Eating Disorder Examination für Kinder (ChEDE)) – körperliche US: Gewicht, Blutabnahme etc. – Psychisches Screening (z.B.SDQ)	Qualitätssicherung	Raum
			Material – Passende Waage, – kleines Labor oder Vernetzung mit Labor …
	Beratung		
	Prävention / Elternarbeit (z.B.Succeat)		IT-Ressourcen
	Behandlungsangebot – Dauer, Frequenz; Evaluation		Informationsmaterial
	Vernetzung – mit Psychotherapeutin – Diätologin		
	Vernetzung mit spezialisierter TK oder Klinik – Zuweisungsprozedere – Rückübernahmeprozedere		
Ambulatorium	Spezialisiertes, evidenzbasiertes Essstörungskonzept	Ambulanz/Ambulatorium	Wieviele spezialisierte Ambulatorien/Region?
	Prävention / Elternarbeit (z.B.Succeat)	Finanzierung (inkl Kooperation)	Raum
	Kooperation mit vor- und nachsorgenden Einrichtungen	Evidenzbasiertes ED-Know-how	Material
	Familienbasierte Betreuung	Teamstruktur – evtl. Hometreatment o. ä.	IT-Ressourcen
		Qualitätssicherung	
		Organisationsentwicklung	
Tagesklinik	Spezialisiertes, evidenzbasiertes Essstörungskonzept	Tagesklinik	Wie viele Tageskliniken/Region/EW?
	Kooperation mit vor- und nachsorgenden Einrichtungen	Finanzierung (inkl Kooperation)	Tagesstruktur
	Familienbasierte Betreuung	Teamstruktur – auf. A. nervosa spezialisiertes Team – multiprofessionelles Team – evtl. Hometreatment o. ä.	Personal
			Material/ IT-Ressourcen
			Essen / Diätologie
			Supervision
		Verwaltung	Fort/Weiterbildung
		Qualitätssicherung	
		Organisationsentwicklung	
Stationär	Notfallversorgung – evtl. in Koop mit Pädiatrie/Interne	Zentralkrankenhaus/Universität	Welche Kliniken werden Zentren?
		Notfallversorgung	Personalstrukturen
	Akutbehandlung – inkl. Akut-Rehaphase	Multiprofessionelles Team	Gebäude und Außenbereiche
		Multi-modales Behandlungsangebot	Material
	Langzeitbehandlung	ED-Zentrums-Know-how	Diätologie/Essen
	Familienbasierte Betreuung	Organisationsentwicklung	Supervision
	Spezialisierung /Schwerpunkt		Fort/Weiterbildung/Forschung/Lehre
	Vernetzung – Zuweisern/Nachsorge – Reha-Zentren		
	Wissenschaft und Lehre		
Spezialisiertes Reha-Zentrum oder Wohneinheiten	Langzeitbehandlung und Rehabilitation	Multiprofessionelles Team	Wie viele spezialisierte Reha-Zentren?
	Familienbasierte Betreuung	Spezialisierung auf ED	Räume
	Vernetzung – Zuweisenden Kliniken – Nachsorge – Niedergelassenen FÄ f KJP	Wohnversorgung	Personal
		Arbeits‑/Schulversorgung	Gebäude und Außenbereiche
		Organisationsentwicklung	Diätologie/Essen
			Supervision/Weiterbildung

Abschließend sei noch erwähnt, dass eine wichtige gesundheitspolitische Maßnahme, nämlich die Schaffung von Mental Health Rehabilitationszentren nicht gemeinsam mit der Versorgungsplanung im ÖSG erfolgt und zusätzlich vom ursprünglichen Mehrebenenmodell abgegangen wurde [23]. Im Sinne des erwähnten Mehr-Ebenen-Modells der KJP-Versorgung ist dringend zu fordern, dass alle Versorgungsangebote unter einer globalen Entwicklungs- + Versorgungsperspektive betrachtet und geplant werden. Zum Vergleich dieser Herangehensweise sei auf Tab. 3 verwiesen, die die ÖSG-Prinzipien der Versorgung definiert.

**Table 3:** 

Prinzip der Bedarfsgerechtheit	Sicherstellen einer dem patientenspezifischen Versorgungsbedarf entsprechenden indizierten ärztlichen und therapeutischen sowie pflegerischen altersgerechten Versorgung inkl. Nahtstellenmanagement zum Sozialbereich
Prinzip der Versorgungsgerechtigkeit	Sicherstellen eines gleichwertigen Zugangs zur Gesundheitsversorgung durch regional möglichst ausgewogene Verteilung der Versorgungsangebote
Qualitätsprinzip	Sicherstellen einer qualitativ hochwertigen Versorgung durch gut ausgestattete und organisierte Versorgungsangebote mit hoher Behandlungsqualität (Berücksichtigung entscheidender Faktoren wie z. B. ausreichende Routine durch Mindestfallzahlen, sachgerechtes Backup), Bündeln von Leistungsangeboten, um bestmögliche Ergebnisse zu erzielen
Effektivitätsprinzip	Sicherstellen einer zum Nachfragezeitpunkt erforderlichen therapierelevanten Diagnostik und darauf aufbauender Behandlung und Betreuung, um Gesundheit und Lebensqualität von PatientInnen zu erhalten, wiederherzustellen oder zu verbessern
Effizienzprinzip	Sicherstellen der Leistungserbringung mit adäquatem Ressourceneinsatz und Nutzung von Synergien (Berücksichtigung entscheidender Faktoren wie z. B. Kontakte bzw. Frequenzen (➜ *Glossar*) und Anzahl erbrachter Leistungen, Mindestauslastung teurer Infrastruktur und spezialisierter Versorgungs-teams; abgestufte Versorgung und Vernetzung durch Kooperationen intra- und extramural)
Ökonomieprinzip	Berücksichtigung von gesamtwirtschaftlichen Auswirkungen und Finanzierbarkeit der geplanten Versorgungsangebote
Prinzip des „Best Point of Service“	Sicherstellen, dass die jeweils richtige Leistung zum richtigen Zeitpunkt am richtigen Ort mit der optimalen medizinischen und pflegerischen Qualität gesamtwirtschaftlich möglichst kostengünstig erbracht wird

Die Prinzipien sind gut definiert, allerdings die dazugehörigen Kriterien fehlen, dazu nur ein Beispiel: wo genau ist der „best point of service“ in unserem Fach und woran wird wie, von wem, was gemessen, analysiert, verglichen und dann in ein Ergebnis umgesetzt?

Zuletzt soll noch festgehalten werden, dass die gesamte Versorgungsplanung, zumindest für die KJP von einem deutlich Defizit-orientierten Modell ausgeht und es hoch an der Zeit wäre, Modelle zu entwickeln, die sich der Gesundheitsdefinition der WHO annähern und dem generellen Wohlbefinden der Kinder und Jugendlichen widmen und diese auch in die Planungen einbinden. Dieses sollte dann Grundlage der Planung von Versorgungsstrukturen sein und diese danach ausgerichtet werden, wo sie die besten Wirkung in Bezug auf das generelle Wohlbefinden entfalten können.

## Zusammenfassung

Die Kinder- und Jugendpsychiatrische Versorgung ist in den vorhanden Gesetzen klar definiert, es existieren Versorgungsvorgaben hinsichtlich der Menge und der Strukturqualität. Bei genauerer Betrachtung dieser Vorgaben fehlen ganz wesentliche Merkmale kinder- und jugendpsychiatrischen Arbeitens, wie Kooperation, Multimodalität – und -professionalität als zentrale fachdefinierende Merkmale. Weiters wird in der Versorgungsregelung nicht auf alters- oder krankheitsspezifische Aspekt eingegangen, der systemische Aspekt wird nur am Rande als Strukturqualität (Eltern-Kind-Betten) vorgegeben, inhaltlich nicht darauf eingegangen (z. B. Arbeit mit dem Herkunftsmilieu). Weiters fehlen wesentliche Beschreibungen hinsichtlich spezifischer Qualitätsmerkmale, die eine Qualitätssicherung und entsprechendes Benchmarking erlauben würden. Insgesamt würde man sich eine KJP-spezifische Diskussion über das Mehrebenenversorgungsmodell [6] erwarten, die Übergänge zwischen den Versorgungsebenen in beide Richtungen planen und strukturell abbilden. Darüber hinaus sollte es eine zentrale Zuständigkeit geben für alle Bereiche der Mental Health bei Kindern und Jugendlichen mit dem Pouvoir Ressort- und Sektorenübergreifend planen und handeln zu können („mental health in all policies“). Es fehlen auch Spezialisierte Zentren (z. B. für Essstörung, Autismus u. v. m.). Dringend müssen auch moderne Konzepte der Versorgung eingebracht und entwickelt werden, wie telemedizinische Möglichkeiten (z. B. [24]), Home-Treatment [25] und Integrative Versorgungsmodelle [26] oder Transitionsmodelle [27], wie dies eigentlich im ÖSG 2017 [8] bereits vorgesehen ist (siehe Abb. 3).
